# Association of oxidative status and semen characteristics with seminal plasma proteins of buffalo semen

**Published:** 2016

**Authors:** L. Sharma, V. Pandey, R. Nigam, A. Saxena, D. K. Swain, B. Yadav

**Affiliations:** 1 Department of Veterinary Biochemistry, College of Veterinary Science and Animal Husbandry, Veterinary University, Mathura, 281001, India;; 2 Department of Veterinary Obstetrics and Gynecology, College of Veterinary Science and Animal Husbandry, Veterinary University, Mathura, 281001, India;; 3 Department of Veterinary Physiology, College of Veterinary Science and Animal Husbandry, Veterinary University, Mathura, 281001, India

**Keywords:** Buffalo, Oxidative status, Season, Semen characteristics, Seminal plasma protein

## Abstract

To study the influence of season on oxidative status of buffalo semen and their association with semen characteristics and seminal plasma proteins, ejaculates were collected twice a week in winter, summer and rainy seasons from six adult Bhadawari buffalo bulls. The neat semen was analyzed for semen characteristics immediately after collection and oxidative status viz. lipid peroxidation (LPO), catalase (CAT), super oxide dismutase (SOD) activity, and total protein (TP) were estimated in seminal plasma. The protein profiling was carried out by one-dimensional sodium dodecyl sulphate polyacrylamide gel electrophoresis (SDS-PAGE). The significant effect of season was observed on TP, SOD activity and % protein fractions of seminal plasma proteins of buffalo bulls. The TP values showed positive correlation with ejaculate volume (EV), sperm concentration (SC), and % live-dead (LD) and negative correlation with progressive motility (PM), and hypo-osmotic swelling test (HOST). The SOD activity showed positive correlation with PM, LD, HOST and % acrosoamal integrity (AI). Besides that, results showed correlation of TP with 6.5, 38 and 66 kDa proteins, LPO with 70, 72, 84 and 86 kDa proteins, CAT with 70 kDa and 86 kDa proteins, and SOD with 6.5, 24.5, 44.5, 70 and 72 kDa proteins. In conclusion, this study indicated that TP and SOD activity of seminal plasma of buffalo bulls were influenced by season and were found to be associated with some of the semen characteristics and expression of seminal plasma proteins.

## Introduction

In tropical and sub-tropical countries, climatic heat is a problem of great concern affecting the growth, productivity and fertility of animals. Scarcely distributed sweat glands and dark body colour of buffalo make them prone to physical distress compared to other farm animals when they are exposed to high ambient temperature and cause oxidative stress *in vivo*, which is reflected as an imbalance between production of reactive oxygen species (ROS) (Kowalowka et al., 2007 ▶) and prompt detoxification of these reactive intermediates by antioxidant system (Hampl et al., 2012 ▶).

The sperms are extremely vulnerable to oxidative attack (Saleh and Agrawal, 2002 ▶) by ROS owing to high content of polyunsaturated fatty acids (PUFA) in their membrane (Lenzi et al., 2000 ▶). Lipid peroxidation (LPO) of these PUFA by ROS can lead to diminished sperm membrane fluidity, loss of membrane integrity, increase in cell permeability leading to enzyme inactivation and structural damage of DNA that may result in impaired sperm function and even cell death (Agarwal and Saleh, 2002 ▶). In addition, low concentrations of scavenging enzymes in mature spermatozoa reduce their capacity for repairing oxidative damage (Alvarez and Storey, 1989 ▶). However, seminal plasma is reported to be endowed with non-enzymatic and enzymatic antioxidant capacity which is provided by the epididymal epithelium (Jimenez et al., 1990 ▶). The semen of different species is reported to contain super oxide dismutase (SOD), glutathione reductase (GR), glutathione peroxidase (GPx) and catalase (CAT) (Alvarez and Storey, 1989 ▶) antioxidant enzyme system (Martí et al., 2007 ▶).

The reproductive performance of breeding bulls is mainly influenced by macro and micro climatic factors of season such as ambient temperature, humidity, rainfall, photo-period (Mandal et al., 2000 ▶) and render significant effect on sexual activities as well as seminal attributes (Sharma et al., 2014 ▶). Thus estimation of bio-chemical parameters of seminal plasma along with semen characteristics becomes necessary for satisfactory semen appraisal in the current practice of commercial artificial insemination (Pandey et al., 2014a ▶, b ▶). Keeping in mind the above said points, an attempt was made to assess the influence of season on oxidative status in buffalo semen and their association with seminal plasma proteins as well as semen characteristics.

## Materials and Methods

**Animals**

 Six sexually mature Bhadawari buffalo bulls (2-4 years old) maintained in nearly identical nutritional and management conditions were selected from Dairy Farm of College of Veterinary Science. Geographically this area is situated in a semi arid zone of northern India (27 in Latitude and 78 Longitude) and 160 meters above sea level with average daily temperature and humidity in a season as depicted in Table 1. The period of study was divided into rainy season (July to September), winter season (December to February) and summer season (April to June) as per conditions prevailing in the area of investigation. To get the specific effect of season on spermatogenesis, the semen samples were selected in the last month of the selected season, usually after 50-60 days of the season had elapsed.

**Semen characteristic evaluation**

Ejaculates were collected twice a week from each bull in morning hours using sterilized artificial vagina and immediately after collection physiological semen attributes were analyzed such as ejaculate volume (EV), mass motility (MM), sperm concentration (SC), progressively motility (PM), and sperm morphology viz. % live-dead (LD) spermatozoa, % hypo-osmotic swelling test (HOST), and % acrosomal integrity (AI) using standard methods (Sharma *et al*., 2014). The seminal plasma was harvested by centrifugation at 5000 rpm for 10 min at 4°C and the supernatants were separated in Eppendorf tubes and stored at -20°C until used.

**Biochemical assays**

Seminal plasma samples were analyzed for the estimation of total protein (TP) by spectrophotometric method at 280 nm, LPO in sperm cell membrane in terms of malondialdehyde (MDA) production by thiobarbituric acid assay method (Rehman, 1984 ▶), SOD by the method described by Madesh and Balasubramanian (1998) ▶ and CAT by the method described by Bergmayer (1983). ▶

**Evaluation of molecular weight of seminal plasma proteins**

In seminal plasma, the protein extraction was carried out using Triprep extraction kit (Fisher Scientific) as per the kit. Protocol recovered proteins were re-suspended in phosphate buffered saline (PBS) and stored at -20°C until analysed. The recovered protein samples from semen of individual animals of each season were pooled and subjected to 12% SDS-PAGE (Laemelli 1970 ▶). A broad range molecular weight marker (Merck, Germany) was run along with protein samples to determine the relative molecular weights of seminal plasma proteins.

**Image analysis and statistical analysis**

 Gel images were analyzed to determine molecular weights of protein bands and relative protein fractions (protein %) using the gel documentation and analysis system (Gel-Doc. Model-Alpha Imager TM1220, Alpha Innotech Corporation, USA). Statistical significance was determined by an ANOVA followed by Tukey’s post-hoc multiple comparison test using SPSS software for windows (version 16.0). Pearson’s correlation analysis was conducted to investigate the association between variables. The data are presented as the mean ± SE and a p-value <0.05 was considered to be statistically significant.

## Results

**Fig. 1:**
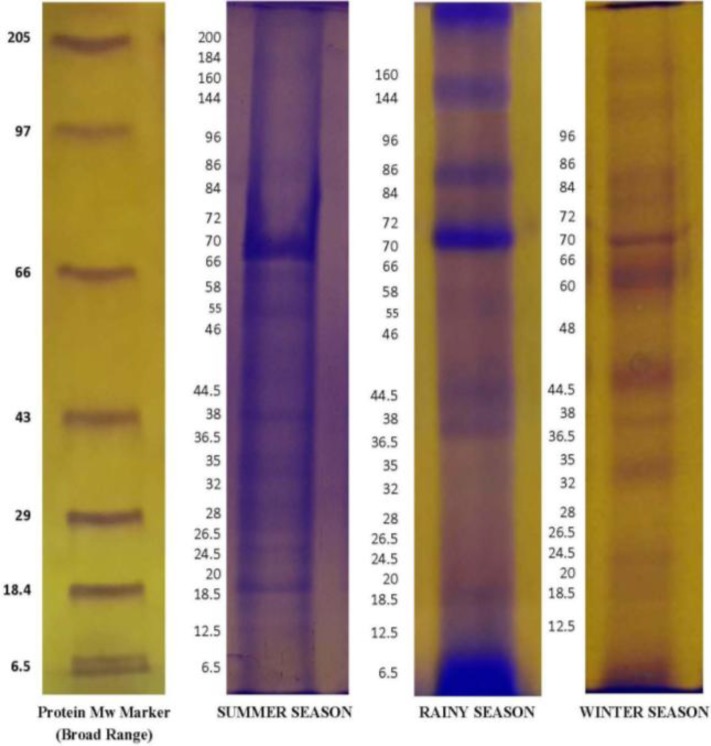
SDS-PAGE protein profile of seminal plasma of Bhadawari buffalo bull semen in three different seasons (viz. summer, rainy, winter)

The mean values of biochemical indices of buffalo seminal plasma are presented in Table 2. The results revealed significant effect of season on TP and SOD activity. Total protein showed the highest concentration in summer season compared to other season whereas SOD activity revealed the highest activity in summer months and the lowest in winter months. The significant effect of season was not observed on CAT activity and extent of LPO. Besides, the results showed significant effect of season on semen characteristics (Sharma et al., 2014 ▶) and seminal plasma protein profile (Fig. 1).

The correlation results of oxidative status and semen characteristics are presented in Table 3. Total protein showed positive correlation with CAT, EV, SC and LD while negative correlation with PM and HOST. Super oxide dismutase activity revealed positive correlation with PM, LD, HOST and AI. Though CAT did not show seasonal variation, it revealed positive association with TP, EV and SC.

The EV showed positive correlation with MM, SC, and LD and negative correlation with HOST while MM revealed positive association with EV and SC. The SC revealed positive association with EV, MM, and LD whereas it was negative with HOST. The LD showed positive correlation with EV, AI, and SC while AI showed positive correlation with PM, LD, and HOST. Positive correlation was observed for HOST with PM and AI but negative with EV and SC.

**Table 1:** Seasonal variations in environment temperature (ºC) and relative humidity (%)

Parameters	Rainy season	Winter season	Summer season
Environment temperature (°C)	29.47 ± 0.07^a^	12.15 ± 0.63^b^	35.16 ± 0.13^c^
Relative humidity (%)	80.77 ± 0.70^a^	83.38 ± 0.83^b^	49.66 ± 1.30^c^

Means bearing at least one common superscript in one parameter did not differ significantly (P≥0.05), otherwise significant at 5% level (P<0.05)

**Table 2:** Seasonal variation in oxidative status of seminal plasma of Bhadawari buffalo bull semen (Mean±SE

Parameters	Rainy season	Winter season	Summer season	Overall mean
Total protein (gm/dl)	3.70 ± 0.19^a^	6.19 ± 0.25^b^	7.33 ± 0.11^c^	5.74 ± 0.18
Lipid per oxidation (nano moles MDA/ml)	2.90 ± 0.04^a^	2.84 ± 0.20^a^	2.97 ± 0.23^a^	3.11 ± 0.11
Catalase activity (mM H_2_O_2_ utilised/min/mg of protein)	23.36 ± 0.25^a^	24.25 ± 0.30^a^	24.22 ± 0.30^a^	22.81 ± 0.22
Super oxide dismutase activity (U/mg of protein)	42.69 ± 0.40^b^	37.35 ± 0.65^a^	44.92 ± 0.56^c^	41.65 ± 0.44

Means bearing at least one common superscript alphabet in one parameter did not differ significantly (P≥0.05), otherwise significant at 5% level (P<0.05)

**Table 3:** Correlation coefficient (r^2^ value) between oxidative status and semen characteristics of Bhadawari buffalo bull semen

Semen characteristics	TP	LPO	CAT	SOD
Total protein (TP)	1.00	0.08	0.05	0.30**
Lipid peroxidation (LPO)	0.08	1.00	-0.02	0.06
Catalase (CAT)	0.05	-0.02	1.00	0.07
Super oxide dismutase (SOD)	0.30**	0.06	0.07	1.00
Ejaculate volume (EV)	0.51**	0.04	0.05	0.19*
Mass motility (MM)	0.13	-0.15	0.04	-0.14
Sperm concentration (SC)	0.60**	-0.11	0.05	0.21*
Progressive motility (PM)	-0.39**	0.03	-0.15	0.32**
% Live-Dead sperm (LD)	0.39**	0.00	0.05	0.41**
% HOST	-0.38**	-0.11	-0.12	0.35**
% Acrosomal integrity (AI)	-0.15	0.08	-0.13	0.39**

* Significant at 5% level, and

** Significant at 1% level

The correlations between oxidative status and seminal plasma proteins are depicted in Table 4. The TP showed positive correlation with 6.5 and 66 kDa and negative with 38 kDa proteins. The SOD activity showed positive correlation with 6.5, 24.5, 44.5, 70 and 72 kDa proteins. The LPO values exhibited negative correlation with 70, 72 and 84 kDa proteins though a positive correlation with 86 kDa protein. The CAT activity revealed positive correlation with 70 kDa and negative correlation with 86 kDa proteins.

**Table 4:** Correlation coefficient (r^2^ value) between oxidative status and protein profile fraction of Bhadawari buffalo bull semen

Protein (kDa)	TP	LPO	CAT	SOD
6.5	0.51*	-0.06	-0.13	0.88**
12.5	-0.35	-0.15	-0.14	0.22
18.5	0.09	0.40	0.40	0.41
20	-0.02	-0.27	-0.35	-0.46
24.5	-0.27	0.41	0.21	0.58*
26.5	0.05	-0.32	-0.27	-0.09
28	0.26	0.17	0.45	0.03
32	-0.11	-0.36	-0.36	-0.11
35	-0.11	0.16	0.22	0.21
36.5	0.07	0.12	0.38	0.39
38	-0.47*	-0.05	-0.04	-0.05
44.5	0.17	0.45	0.12	0.53*
66	0.60**	-0.15	0.05	0.14
70	0.21	-0.70**	0.57*	0.50*
72	0.05	-0.68**	0.44	0.61**
84	0.17	-0.53*	-0.28	-0.28
86	-0.07	0.54*	-0.60**	-0.33
96	-0.27	-0.24	-0.17	-0.15

* Significant at 5% level, and

** Significant at 1% level

## Discussion

The significant influence of season was observed on semen characteristics viz. EV, SC, PM, LD, HOST, and AI as well as on seminal plasma proteins profile in the same study published earlier (Sharma et al., 2014 ▶). The present study showed significant effect of season on seminal plasma TP concentration and SOD activity. The semen of summer season showed higher TP compared to winter and rainy season. The seasonal variation in TP and composition of SP was also reported in buffalo (Khawaskar et al., 2012 ▶) and ram (Marti et al., 2007 ▶). However, Nandre (2008) ▶ and Khawaskar et al. (2012) ▶ reported higher protein concentration in seminal plasma in winter compared to summer season in Surti buffalo.

The higher seminal plasma TP could exert protective effects on the sperm plasma membrane integrity, which maintains the sperm function. Similarly, Cardozo et al. (2006) ▶ reported that impaired sperm viability in spring or summer in rams may be due to reduced protein content in the seminal plasma. Furthermore, Strzezek et al. (1999) ▶ reported significant correlation between TP content and antioxidant properties of the seminal plasma. The findings of present study reaffirm that seminal plasma protein plays an important role in sperm functions, corroborating the present findings.

The SOD activity in seminal plasma of buffalo showed significant higher values in summer season than other seasons in this study. Similar higher values of SOD activity were observed by Shek-Vugrovecki et al. (2011) ▶ in Simmental bull semen, corroborating the observations of present study. However, influence of season was not observed on SOD activity in semen of *Bos taurus* bulls (Nichi et al., 2006 ▶). This higher activity of SOD may be associated with high SC and better semen quality as significant correlation between SOD activity and SC was reported by Hammadeh et al. (2009) ▶. The influence of season was not observed on CAT activity and LPO of buffalo semen in this study. But there was tendency of higher values of CAT activity in winter and LPO in summer season compared to other seasons. Likewise Marti et al. (2007) ▶ and Shek-Vugrovecki et al. (2011) ▶ also reported non-significant change in MDA levels and CAT activity in seminal plasma of ram and Simmental bulls, respectively, corroborated the findings of present study. The similar higher LPO in autumn and summer as compared to winter season that was observed by Nichi et al. (2006) ▶ in Simmental bulls mirrors the present study.

Under normal circumstances, there is an appropriate balance between oxidants and antioxidants and this balance between production and disposal of oxidant molecules is essential for spermatozoa homeostasis. The increased rate of free radicals production or decreased rate of their removal by low levels of SP antioxidants leads to free radical accumulation causing lipid peroxidation of membrane leading to cellular damage (Berry and Kohen, 1999) and infertility (Chen et al., 2001 ▶).

The correlation among oxidative status and semen characteristics showed positive correlation of TP with EV, SC, LD and was negative with SOD. Kumar et al. (1984) ▶ and Khawaskar et al. (2012) ▶ also reported association of TP with these semen parameters in Murrah and Surati buffaloes, respectively simulates the findings of present study. Similarly, negative association between SOD and TP was also observed by Selvaraju et al. (2010 ▶) in Surti buffalo which substantiates the findings of this study.

Super oxide dismutase activity of seminal plasma showed positive correlation with EV, SC, PM, LD, HOST, AI, and TP in present study. Kadirvel et al. (2014) ▶ also reported positive correlation of SOD with SC, PM, and livability in bull and buffalo seminal plasma, which simulate the reports of present study. Results of this study suggest that higher levels of antioxidant prevent lipid peroxidation in spermatozoa and therefore result in higher sperm motility. The positive correlations between seminal SOD and SC may be interpreted similar to Hsieh et al. (2002) ▶ who suggested that higher concentrations of spermatozoa might produce higher levels of SOD (Shamsi et al., 2009 ▶). In present study, the higher activity of SOD observed in summer season may be associated with high SC and better semen quality as significant correlation between SOD activity and SC was also reported by Hammadeh et al. (2009) ▶.

The extent of LPO and CAT activity did not show significant correlation with semen characteristics and oxidative status. Similarly, Shamsi et al. (2009) ▶ reported positive but non-significant correlation of CAT with SC which substantiates the findings of present study. In fact, Atig et al. (2012) ▶ revealed a significant negative correlation between LPO and sperm motility.

The AI showed association neither with semen characteristics nor with oxidative status which is in agreement with the reports of Kadirvel et al. (2014) ▶. These results are in line with Baumber et al. (2000) ▶ and Neild et al. (2005 ▶), who reported that the acrosomal damage was independent from ROS and lipid peroxi-dation. It seems that the acrosomal damage during different season might be stress-related.

The parameters of oxidative status showed correlation of TP with 66 kDa proteins, LPO with 70, 72 and 86 kDa proteins, CAT with 70 and 86 kDa proteins and SOD with 70 and 72 kDa proteins. Scarce literature is available with which we can compare the results of correlation between protein fractions with oxidative status. However, Marti et al. (2007) ▶ reported some kind of association between 32.5 and 88 kDa proteins with SOD, while none of the eluted fractions showed CAT activity. In addition, de Souza et al. (2006) ▶ identified 37 proteins in seminal plasma of dogs; two bands, B4 (67 kDa) and B5 (58.6 kDa), were found significantly correlated with sperm motility (r=0.66 and r=0.46), and the HOST (r=0.76 and r=0.68), respectively. These findings are partially in agreement with the results of present study.

It is noteworthy that although some protein bands did not show seasonal variations in their protein content, they were found to correlate with some seminal quality and oxidative parameters. The obtained correlation coefficients were also not very high for proteins with or without seasonal changes. These findings suggest that proteins in the seminal plasma would act in a complementary manner, and that these proteins play an important role in sperm membrane stability, and subsequent viability, motility and concentration.

Furthermore, although we detected significant correlations among oxidative status, seminal plasma proteins and semen characteristics, it is noteworthy that correlation does not imply cause. Therefore, these findings should be interpreted with caution, pending further studies that directly relate seminal plasma proteins and fertility. In addition, more sophisticated studies that allow higher-resolution separation of seminal plasma proteins and more detailed characterization of those proteins, as well as investigation of their physiological role, will further advance knowledge in this area.
